# TomatoDet: Anchor-free detector for tomato detection

**DOI:** 10.3389/fpls.2022.942875

**Published:** 2022-08-05

**Authors:** Guoxu Liu, Zengtian Hou, Hongtao Liu, Jun Liu, Wenjie Zhao, Kun Li

**Affiliations:** ^1^Goertek College of Science and Technology Industry, Weifang University, Weifang, China; ^2^School of Intelligent Manufacturing, Weifang University of Science and Technology, Weifang, China; ^3^Weifang Key Laboratory of Blockchain on Agricultural Vegetables, Weifang University of Science and Technology, Weifang, China; ^4^School of Computer, Weifang University of Science and Technology, Weifang, China

**Keywords:** tomato detection, anchor-free, CenterNet, deep learning, harvesting robots

## Abstract

The accurate and robust detection of fruits in the greenhouse is a critical step of automatic robot harvesting. However, the complicated environmental conditions such as uneven illumination, leaves or branches occlusion, and overlap between fruits make it difficult to develop a robust fruit detection system and hinders the step of commercial application of harvesting robots. In this study, we propose an improved anchor-free detector called TomatoDet to deal with the above challenges. First, an attention mechanism is incorporated into the CenterNet backbone to improve the feature expression ability. Then, a circle representation is introduced to optimize the detector to make it more suitable for our specific detection task. This new representation can not only reduce the degree of freedom for shape fitting, but also simplifies the regression process from detected keypoints. The experimental results showed that the proposed TomatoDet outperformed other state-of-the-art detectors in respect of tomato detection. The F_1_ score and average precision of TomatoDet reaches 95.03 and 98.16%. In addition, the proposed detector performs robustly under the condition of illumination variation and occlusion, which shows great promise in tomato detection in the greenhouse.

## 1. Introduction

Tomato harvesting is a labor-intensive work, which needs a lot of human resources. It is also very time consuming and includes much tedious work. However, with the development of urbanization and aging of society, the people in the countryside have decreased a lot, and the labor cost continues to increase, resulting in a big labor shortage in farming work (Yue et al., 2015). On the other side, intelligent agriculture is developing fast in the past decades, which is an ideal substitute of human resources for farming work. Among the various technologies applied in the agriculture, the fruit harvesting robot is one of the prominent artificial intelligent techniques. It has huge potential efficiency in fruit harvesting, which can bring high profit as well as liberating the labor force. Thus, it is of great value and significance to develop harvesting robots.

A harvesting robot usually consists of two components—a vision system and an eye-hand coordination system (Zhao et al., 2016a). The vision system plays a key role in the whole system, since the first critical step for the harvesting robot is to detect fruits autonomously. This step determines the detection and subsequent picking accuracy of harvesting robots. Thus, it is very crucial to develop a robust fruit detection algorithm of the vision system. However, at present, no harvesting robot has been commercialized successfully due to either low detection accuracy or low detection speed. Many factors have hindered the pace of harvesting robot development such as uneven illumination, occlusion, overlap, and some other unpredictable factors (Gongal et al., 2015).

To deal with the above challenges, many researchers have studied fruit detection over the past years. In the early years, some researchers used threshold discriminant methods for fruit detection based on color, shape, texture, or fusion of them (Linker et al., 2012; Kelman and Linker, 2014; Wei et al., 2014), and achieved reasonable detection results. Bulanon et al. (2002) used an optimal threshold extracted from the intensity histogram of a red-color-difference enhanced image for apple recognition. The results showed that the success rate exceeds 88%. This method is restricted to ripe apples which present different color to the background. Okamoto and Lee (2009) employed hyperspectral imaging for detection of green citrus. The method is separated into pixel-wise segmentation process using pixel discrimination functions and fruit recognition process with thresholds selected by trial and error. This method greatly relies on the selection of several optimal thresholds, and thus is lack of robustness when the fruit environment changes. Inspired by the eigenface concept, Kurtulmus et al. (2011) proposed a novel eigenfruit feature for green citrus detection, combined with color and circular gabor texture. Although intrinsic texture features are used other than only color features, the method still confuses some fruits with background and does nothing with severe occluded fruits. Zhao C. et al. (2016) developed a cascaded pixel segmentation method for immature citrus detection in natural environment. Three color feature maps and a block matching method are adopted to identify potential fruit pixels. Finally, an SVM classifier is used to remove false detections. Nevertheless, with only color feature for segmentation in the early stage, many fruits are missed by the method due to similarity between green fruits and background. Zhao et al. (2016b) proposed a multi color feature fusion method based on wavelet transformation for mature tomato recognition. The detection accuracy reaches 93%. However, since only color features are employed, the method is inferred to be sensitive to illumination variation. These methods greatly rely on the selection of suitable thresholds, making them sensitive to the changes in the form of fruit presentation, such as illumination variation and occlusion.

With the development of machine learning, many researchers tried to apply them to fruit detection, such as adaboost, support vector machine (SVM) or other statistical classifiers (Kurtulmus et al., 2014; Lv et al., 2014; Yamamoto et al., 2014), and get better results than the threshold discriminant methods. Zhao et al. (2016c) used an adaboost classifier associated with haar features for tomato detection. An average pixel value feature is adopted for the removal of false detections. More than 96% of tomatoes are detected in their study. Li et al. (2017) proposed to use an SVM trained on histogram-based features for green and ripe tomato recognition. Prior to detection, the fast normalized cross correlation method is used to extract the potential tomato regions. Finally, the circular hough transform and color analysis are combined to obtain tomato positions. Behroozi-Khazaei and Maleki (2017) proposed to use an artificial neural network optimized by genetic algorithm for grape cluster detection. Also, the genetic algorithm is adopted for color feature selection, which subsequently serves as input to the network. A Bi-Layer schema was proposed for automatic detection of ripening tomatoes by Wu et al. (2019). In their method, a weighted relevance vector machine is used for tomato recognition based on six color-related features and five textural features. A detection rate of 94.90% is reported in the results. Liu et al. (2019) developed a coarse-to-fine method for ripe tomato detection in the greenhouse. First, a naïve bayes classifier is used to identify potential tomato area, on which an SVM classifier combined with histogram of oriented gradients is applied to recognize tomatoes. At last, a color analysis method is proposed to remove false detection. The machine learning methods usually achieve better performance than threshold discriminant methods. However, the low-level abstraction capabilities of hand-crafted features make it difficult to adapt these methods to complicated environmental change.

The emergence of deep learning methods especially convolutional neural networks provides a new paradigm for computer vision tasks, including fruit detection tasks (Sa et al., 2016; Tian et al., 2019; Zheng et al., 2021). These methods can learn feature representations directly from the data and can be trained end-to-end. Nevertheless, the detection accuracy and robustness still need to be improved to enable real commercial applications under complicated conditions as discussed above.

**Figure 1:**
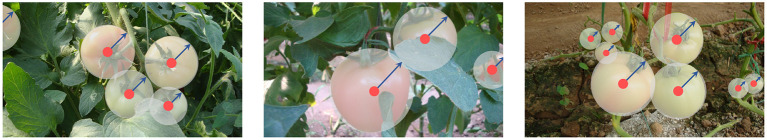
A tomato is modeled as a center point of its bounding circle. The radius of the bounding circle can be inferred from the keypoint at the center.

To address the above problems, this study proposes an effective anchor-free detector called TomatoDet for tomato detection. The proposed model represents a tomato by the center point of its bounding circle, as shown in Figure 1. First, to improve the expression ability of the backbone network, an attention mechanism is introduced to guide the network to pay more attention to the region of interest (ROI), especially small tomatoes. Second, a bounding circle is adopted for tomato localization instead of the traditional bounding box, which is commonly used for general object localization.

Our main contribution is three-fold as follows:

1. The Convolutional Block Attention Module is introduced into the backbone network of CenterNet (Zhou et al., 2019) called Attentive-DLA34 to boost the representation power.
2. A circle representation for tomato detection is adopted to adapt the traditional detection methods to our specific detection task. The new circle representation not only reduces the degree of freedom for shape fitting, but also simplifies the regression process from detected keypoints.
3. Extensive experiments are conducted on tomato datasets. We show that the proposed TomatoDet achieves better performance in terms of both accuracy and robustness, compared to the original CenterNet and other state-of-the-art object detectors.

## 2. Related work

In recent years, deep learning methods have shown continuous performance improvements on fruit detection. A “MangoYOLO” detector was proposed for fruit detection and fruit load estimation by Koirala et al. (2019). This model combines the advantages of YOLOv2 (Redmon and Farhadi, 2017) and YOLOv3 (Redmon and Farhadi, 2018), which has both high detection speed and detection accuracy. It outperforms other methods such as Faster R-CNN (Ren et al., 2015), YOLOv2 (Redmon and Farhadi, 2017), YOLOv3 (Redmon and Farhadi, 2018), and SSD (Liu et al., 2016), on their Mango dataset. Bresilla et al. (2019) improved YOLO (Redmon et al., 2016) model for apples and pears detection. First, the grid-scale is scaled up twice to fit the size of the fruits. Second, the model is pruned to improve the detection speed while not degrading the accuracy. Afonso et al. (2020) applied Mask R-CNN to the tomato dataset for detection. Several neural networks are used as backbone for feature extraction. The best F_1_ score reaches over 94% in their report. Liu G. et al. (2020) proposed a YOLO-Tomato for tomato detection based on YOLOv3 (Redmon and Farhadi, 2018). A dense architecture is incorporated to the backbone to facilitate feature reuse, and a circular bounding box is adopted to optimize the non-maximum suppression process. The model achieves a competing performance compared to state-of-the-art detection methods. Zheng et al. (2021) improved YOLOv4 (Bochkovskiy et al., 2020) for green citrus detection. First, the backbone network is trimmed to reduce detection time. Then, a novel Bi-PANet is proposed to fuse features from different layers. With the modifications, the detection accuracy is reported to be 86% on their dataset. Zhang et al. (2021) developed an edge-device oriented lightweight model for fruit detection. The structure of the original CSPNet is lightened to boost detection speed, and a deep-shallow feature fusion model is proposed to enhance the expression ability of the network. Tested on three types of edge devices, the average detection precision reaches 93, 84.7, and 85% for oranges, tomatoes, and apples, respectively. Wei et al. (2022) proposed a green fruit detection model based on D2Det. By incorporating MobileNetV2, feature pyramid networks and region proposal network structure into the original model, the detection accuracy of green fruits in orchard environments was greatly improved. Chen et al. (2022) improved YOLOv4 for the detection of citrus by incorporating an attention mechanism and a depthwise separable convolution module. In addition, a pruning algorithm was applied to remove the influence of irrelevant latent factors of the data.

Although exciting results are achieved by the above methods, there is still much room for optimization of the networks to improve detection performance. Moreover, the above methods are all anchor-based methods, which commonly perform nearly exhaustive anchor classification over the image and have many hyperparameters for anchor design, reducing the detection efficiency.

## 3. Materials and methods

### 3.1. Image acquisition

**Figure 2:**
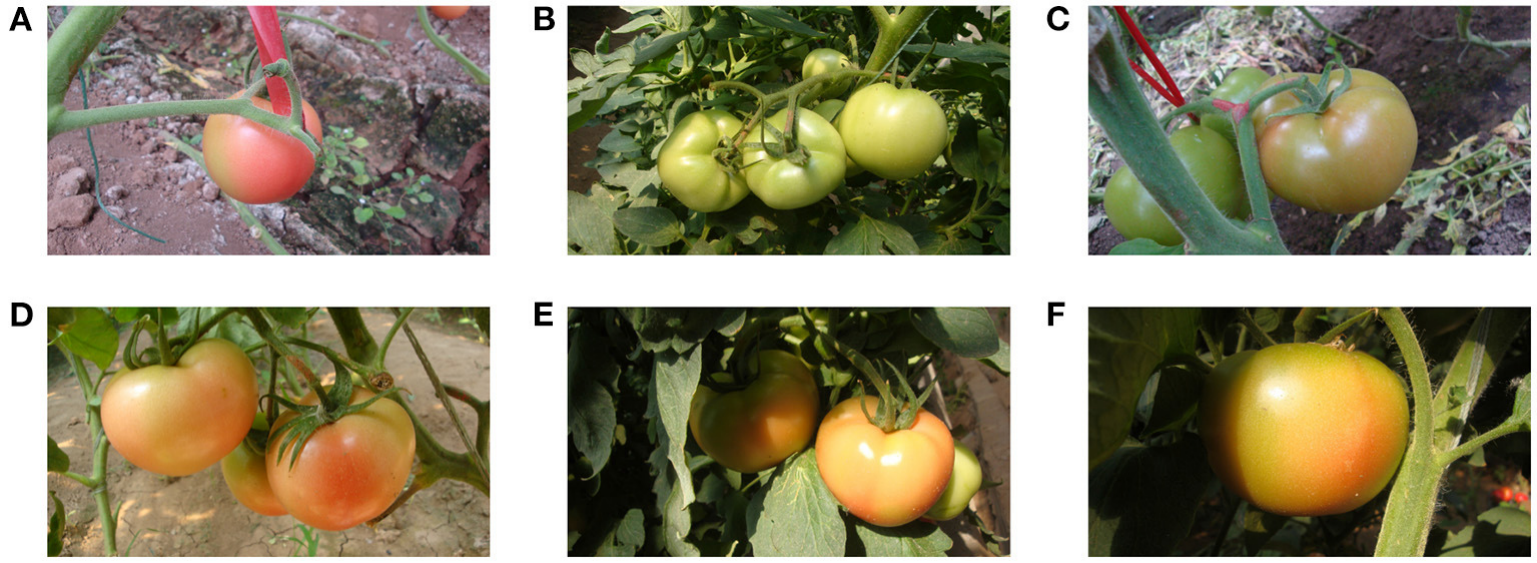
Some tomato samples with different growing circumstances: **(A)** a single tomato, **(B)** a cluster of tomatoes, **(C)** occlusion case, **(D)** overlap case, **(E)** shading case, and **(F)** sunlight case.

The images used in this study are captured using a digital camera (Sony DSC-W170, Tokyo, Japan) with a resolution of 3,648 ×2,056 pixels in a Tomato Production Base, which is located in Shouguang City, Shandong Province, China. The datasets are collected under various environment conditions including sunlight, shading, occlusion, and overlap, etc. Some examples captured under different conditions are shown in Figure 2.

To verify the proposed method, the datasets are split into two subsets—a training set and a test set. The training set contains 725 images, and 241 images are included in the test set. Totally, 966 images are used in this study. For data labeling, a tool called Label-Tomato has been developed to annotate images with proposed bounding circles based on Python. The output format of Label-Tomato is txt files, which include the numbers and locations of tomatoes for each image.

### 3.2. Data augmentation

**Figure 3:**
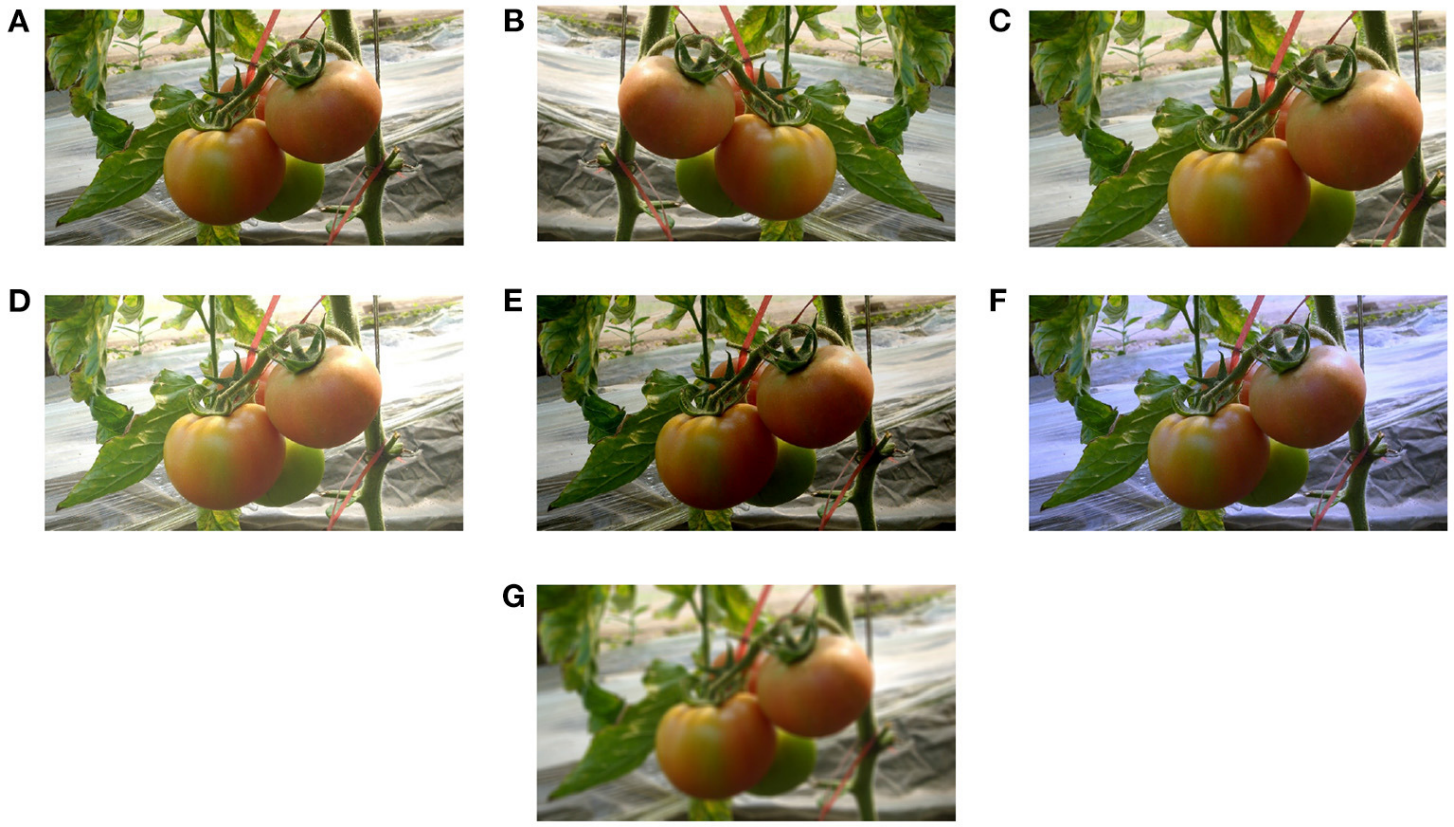
Data augmentation of tomato images: **(A)** original image, **(B)** horizontal flip, **(C)** scaling and cropping, **(D)** high brightness, **(E)** low brightness, **(F)** color balancing, and **(G)** blur processing.

To avoid over-fitting of the model in the training process, the data augmentation is used in this study to simulate real-life interference and enhance the richness of the collected datasets. Several image processing technologies are adopted for augmentation - horizontal flip, scaling and cropping, brightness transformation, color balancing and image blurring, as shown in Figure 3. For the brightness transformation, we use a factor falling in the range [0.6, 1.4] to change the intensity of the pixels in the image randomly. This process can simulate different weather factors on the image intensity. For the scaling and cropping operation, we follow the same process as in Liu G. et al. (2020). To eliminate the effect of lighting on color rendering, we adopt the gray world algorithm (Lam, 2005) for color balancing. Finally, we randomly blur the augmented images by flip, scaling and cropping, brightness transformation, and color balancing to simulate indistinct images caused by camera movement. After data augmentation, the whole number of resultant images is shown in Table 1.

**Table 1:** The number of training images after data augmentation.

	**Original**	**Flip**	**Scaling** **and cropping**	**Brightness**	**Color**	**Blur**	**Total**
No. of tomato images	725	725	725	1,450	725	725	5,075

### 3.3. Overview of tomatoDet

**Figure 4:**
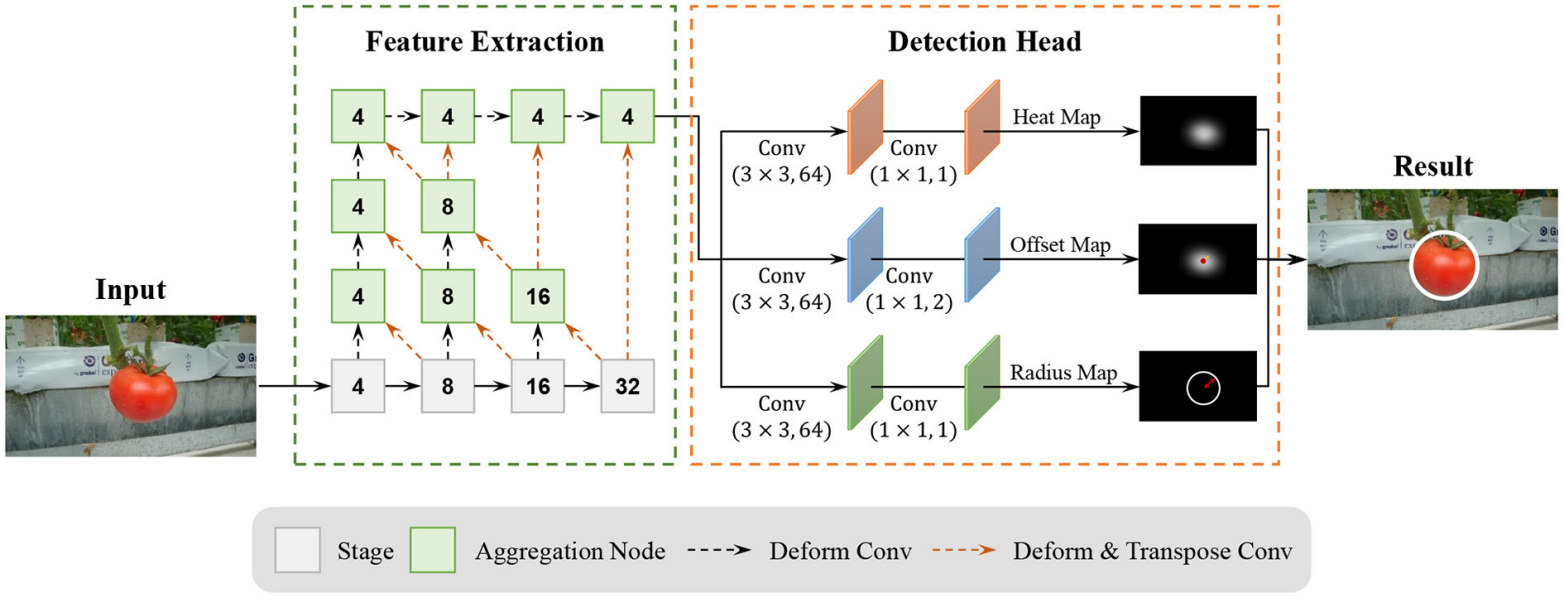
An overview of the proposed model.

Our tomato detection model, called TomatoDet, pools several concepts from the past work with our novel idea to improve the detection performance. An overview of the proposed model is shown in Figure 4. The proposed TomatoDet is based on CenterNet and consists of two modules. The first module is used for feature extraction. It adopts Deep Layer Aggregation-34 (DLA34) (Yu et al., 2018) as the backbone and incorporates Convolutional Block Attention Module (CBAM) (Woo et al., 2018) to improve the feature expression ability and guide the network to focus on small-scale tomato targets. The second module is the detection head. The architecture of the detection head is like that of CenterNet, except that we use a radius head instead of the height and width head for bounding circle prediction. More details are presented in Sections 3.4 and 3.5.

### 3.4. The proposed attentive-DLA34 backbone

**Figure 5:**
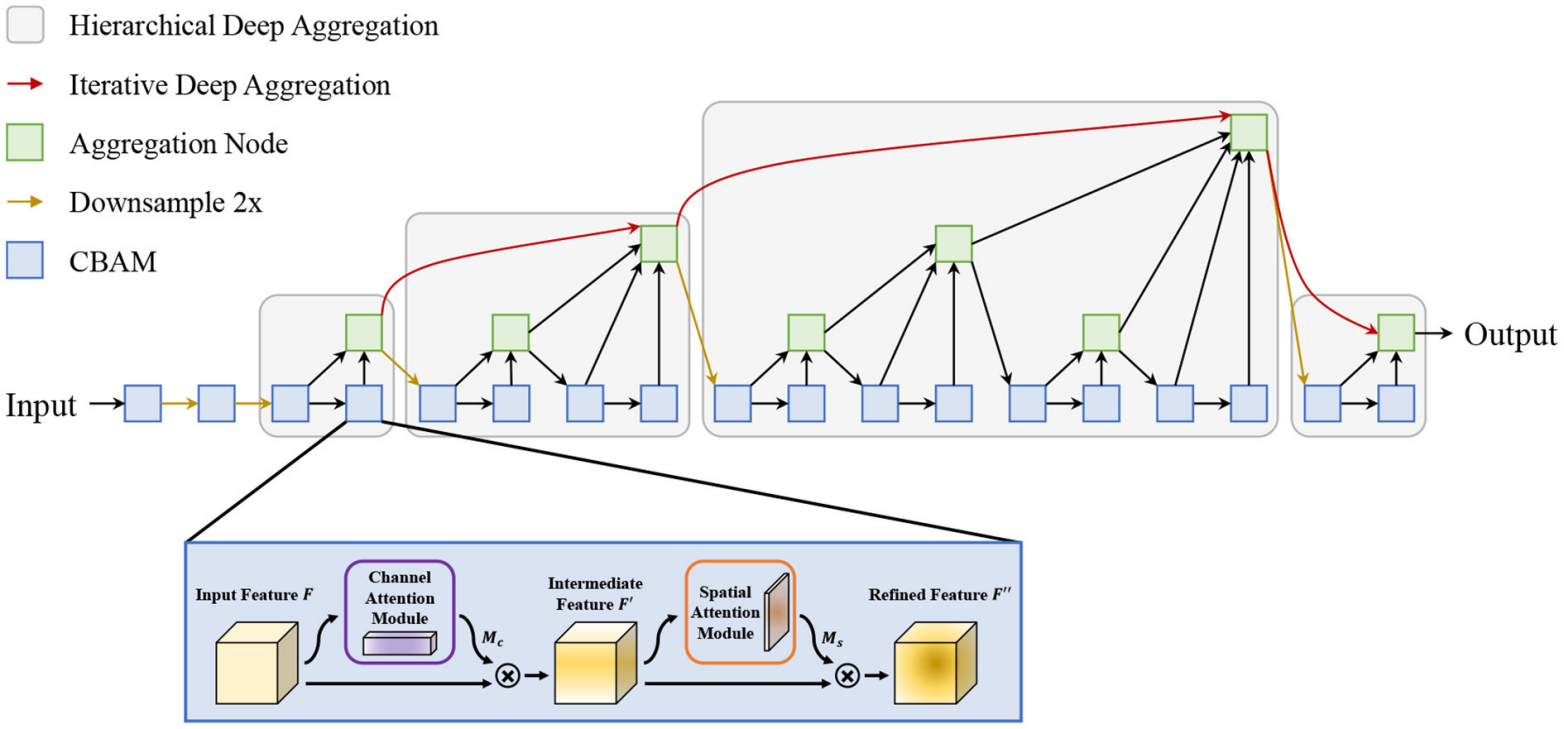
The proposed attentive-DLA34 model.

In this study, an attentive Deep Layer Aggregation network (Attentive-DLA34) is proposed as the base backbone for feature extraction. The DLA is inspired by dense connection and feature pyramid and has two main structures: the iterative deep aggregation (IDA) and the hierarchical deep aggregation (HDA). The IDA is mainly used for feature fusion across resolutions and scales while the HDA focuses on semantic fusion, i.e., aggregating features from different channels and depths in a tree-based structure. Based on these two structures, the DLA could make better use of spatial and semantic information for recognition and localization. However, the complicated conditions make it challenging to detect tomatoes in a natural environment, not to mention the existence of a large number of small tomatoes. To mitigate this problem, we introduce an attention mechanism—Convolutional Block Attention Module (CBAM)—into the backbone network to guide it to pay more attention to the region of interest (ROI). The architecture of the proposed Attentive-DLA34 model is shown in Figure 5.

As shown in Figure 5, we replace the original layers in each stage with CBAM to focus its attention on tomato areas. For CBAM, it is divided into a channel attention module and a spatial attention module in a sequential manner. First, the channel attention module takes the input and infers a 1D channel attention map. Then, the multiplication output of the input and the attention map is inputted to the spatial attention module to get the final output feature map in the same way. The detailed operation can be depicted in Equations (1) and (2):


(1) $$\begin{array}{l} F^{\prime }=M_{c}\left(F\right)⊗F \end{array}$$

(2) $$\begin{array}{l} F^{\prime \prime }=M_{s}\left(F^{\prime }\right)⊗F^{\prime } \end{array}$$

where ⊗ indicates element-wise multiplication, *F*∈*R*^*C*×*H*×*W*^ is the input feature map, $M_{C}\in R^{C\times 1\times 1}$ denotes the generated channel attention map, and $M_{s}\in R^{1\times H\times W}$ denotes the generated spatial attention map. *F*^′′^ is the final output by CBAM.

### 3.5. Circle representation

For general object detection, a bounding box is usually adopted for object localization. However, this type of detection representation is not optimal for specific objects which have a particular shape. In this study, since our detection target is tomato, which is roughly circular, it is better to use bounding circles instead of bounding boxes for localization. It has three folds of advantages. Firstly, compared with bounding boxes, bounding circles could better match the shape of tomatoes. Secondly, the representation of a circle is simpler than that of a box, which makes it easier for the network to learn. Lastly, the circle is invariant to rotation.

#### 3.5.1. From point to bounding circle

For an input image *I* ∈ *R*^*W*×*H*×3^ with width *W* and height *H*, the target is to produce a keypoint heatmap $\hat{Y}\in {\left[0,1\right]}^{\frac{W}{K}\times \frac{H}{K}\times C}$, where *K* is the downsampling ratio of output and *C* is the number of classes. A prediction from the heatmap *Ŷ*_*x, y, c*_ = 1 denotes a detected keypoint, and *Ŷ*_*x, y, c*_ = 0 denotes background. Following Law and Deng (2018), the ground truth of the keypoints is mapped onto a heatmap *Y* using a 2D Gaussian kernel as in Equation (3):


(3) $$\begin{array}{l} Y_{x,y,c}=\exp\left(-\frac{{\left(x-{\tilde{p}}_{x}\right)}^{2}+{\left(y-{\tilde{p}}_{y}\right)}^{2}}{2\sigma_{p}^{2}}\right) \end{array}$$

where ${\tilde{p}}_{x}$ and ${\tilde{p}}_{y}$ are the equivalent groundtruth keypoints of prediction, and they are downsampled by the factor *K* from the original keypoint *p* and are then discretized. σ_*p*_ is a kernel standard deviation.

After getting the peaks of the heatmap for tomatoes, the top *N* peaks are selected among all the detected responses whose value is greater or equal to its eight-connected neighbors. We define $\hat{P}={\left\{\left({\hat{x}}_{i},{\hat{y}}_{i}\right)\right\}}_{i=1}^{N}$ as the set of *N* detected center points. The confidence of the detected bounding circle is represented by the keypoint values *Ŷ*_*x*_*i*_, *y*_*i*_, *c*_, and the center point $\hat{p}$ and radius $\hat{r}$ of the bounding circle is denoted as follows:


(4) $$\begin{array}{l} \hat{p}=\left({\hat{x}}_{i}+\text{Δ}{\hat{x}}_{i},{\hat{y}}_{i}+\text{Δ}{\hat{y}}_{i}\right) \end{array}$$

(5) $$\begin{array}{l} \hat{r}={\hat{R}}_{{\hat{x}}_{i},{\hat{y}}_{i}} \end{array}$$

where $\left(\text{Δ}{\hat{x}}_{i},\text{Δ}{\hat{y}}_{i}\right)={\hat{O}}_{{\hat{x}}_{i},{\hat{y}}_{i}}\in R^{\frac{W}{K}\times \frac{H}{K}\times 2}$ is the offset prediction and ${\hat{R}}_{{\hat{x}}_{i},{\hat{y}}_{i}}\in R^{\frac{W}{K}\times \frac{H}{K}\times C}$ is the radius prediction.

#### 3.5.2. Bounding circle IOU

The intersection-over-union (IOU) is commonly used to evaluate the similarity of two bounding boxes. In this study, we introduce a circle IOU (cIOU) for evaluation of two bounding circles.

**Figure 6:**
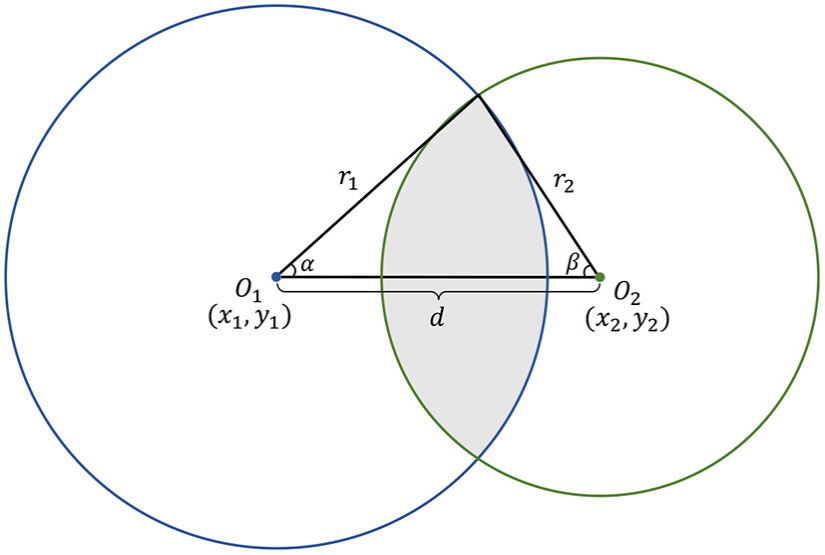
The schematic diagram of cIOU.

As shown in Figure 6, denoting the center coordinates of two intersected circles *O*_1_ and *O*_2_ be (*x*_1_, *y*_1_) and (*x*_2_, *y*_2_), respectively, the distance between two centers *d* can be represented in Equation (6) and satisfies the condition |*R* − *r*| ≤ *d* ≤ |*R* + *r*|.


(6) $$\begin{array}{l} d=\sqrt{{\left(x_{1}-x_{2}\right)}^{2}+{\left(y_{1}-y_{2}\right)}^{2}} \end{array}$$

The angles α and β can be calculated as:


(7) $$\begin{array}{l} \alpha =\cos^{-1}\frac{r_{1}^{2}+d^{2}-r_{2}^{2}}{2r_{1}d} \end{array}$$

(8) $$\begin{array}{l} \beta =\cos^{-1}\frac{r_{2}^{2}+d^{2}-r_{1}^{2}}{2r_{2}d} \end{array}$$

Then, the intersection area *A*_*O*_1_ ∩ *O*_2__ and union area *A*_*O*_1_∪*O*_2__ of circles *O*_1_ and *O*_2_ can be derived as in Equations (9) and (10).


(9) $$\begin{array}{l} A_{O_{1}\cap O_{2}}=\alpha r_{1}^{2}+\beta r_{2}^{2}-\frac{1}{2}r_{1}^{2}\sin2\alpha -\frac{1}{2}r_{2}^{2}\sin2\beta \end{array}$$

(10) $$\begin{array}{l} A_{O_{1}\cup O_{2}}=\pi r_{1}^{2}+\pi r_{2}^{2}-A_{O_{1}\cap O_{2}} \end{array}$$

Consequently, the cIOU can be represented as follows:


(11) $$\begin{array}{l} \mathrm{cIOU}=\frac{\left(2\alpha -\sin2\alpha \right)r_{1}^{2}+\left(2\beta -\sin2\beta \right)r_{2}^{2}}{\left(2\pi -2\alpha +\sin2\alpha \right)r_{1}^{2}+\left(2\pi -2\beta +\sin2\beta \right)r_{2}^{2}} \end{array}$$

### 3.6. Loss function

The loss function of TomatoDet in the training stage consists of three parts, i.e., the keypoint heatmap loss, bounding circle radius loss and center offset loss. The keypoint heatmap loss *L*_*hm*_ is based on focal loss (Lin et al., 2017) as in Equation (12).


(12) $$\begin{array}{l} L_{hm}=\\ -\frac{1}{N}\underset{x,y,c}{\sum }\{\begin{array}{ll} {\left(1-{\hat{Y}}_{x,y,c}\right)}^{\alpha }\log{\hat{Y}}_{x,y,c} & \text{if }Y_{x,y,c}=1\\ {\left(1-Y_{x,y,c}\right)}^{\beta }{\left({\hat{Y}}_{x,y,c}\right)}^{\alpha }\log\left(1-{\hat{Y}}_{x,y,c}\right) & \text{otherwise} \end{array} \end{array}$$

where *N* is the number of keypoints in an image, and α and β are hyper-parameters for the focal loss. In this study, α and β are set to be 2 and 4 following Zhou et al. (2019).

To rectify the keypoint location error resulting from the discretization of downsampling, an offset loss *L*_*off*_ is designed to measure the difference between the predicted offset *Ô* and the groundtruth *O* based on L1 loss.


(13) $$\begin{array}{l} L_{off}=\frac{1}{N}\underset{p}{\sum }\left|{\hat{O}}_{\tilde{p}}-O_{\tilde{p}}\right| \end{array}$$

The tomato radius is regressed from the center points optimized by the radius loss *L*_*r*_ in Equation (14).


(14) $$\begin{array}{l} L_{r}=\frac{1}{N}\overset{N}{\underset{k=1}{\sum }}\left|{\hat{R}}_{p_{k}}-r_{k}\right| \end{array}$$

where ${\hat{R}}_{p_{k}}$ and *r*_*k*_ denotes the predicted and groundtruth radius of the *k*th tomato, and *N* represents the number of results.

Above of all, the total loss of TomatoDet is denoted as in Equation (15).


(15) $$\begin{array}{l} L_{det}=L_{hm}+{\text{λ}}_{off}L_{off}+{\text{λ}}_{r}L_{r} \end{array}$$

where λ_*off*_ = 1 and λ_*r*_ = 0.1 are used in our experiment to balance different losses, referring to Zhou et al. (2019).

### 3.7. Experimental setup

The experiments are performed on a Ubuntu 16.04 with an Intel(R) Core(TM) i7-9700 K CPU@3.60 GHz. It is accelerated by an NVIDIA GeForce GTX 1080Ti GPU. The proposed TomatoDet model is implemented on Pytorch.

The model is trained on an input resolution of 512 ×512 pixels. It is trained with a batch size of 8 and an initial learning rate of 1.25e-4 for 140 epochs. The learning rate is then dropped 10 at 90 and 120 epochs, respectively.

To evaluate the performance of the proposed method, recall (R), precision (P), and F_1_ score are used as the criterion indexes. They are defined in Equations (16)–(18):


(16) $$\begin{array}{l} P=\frac{TP}{TP+FP} \end{array}$$

(17) $$\begin{array}{l} R=\frac{TP}{TP+FN} \end{array}$$

(18) $$\begin{array}{l} F_{1}=\frac{2\times P\times R}{P+R} \end{array}$$

where TP, FP, and FN represent true positives (correct detections), false positives (false detections), and false negatives (missing detections), respectively.

Besides, the average precision (AP) is adopted in this study to evaluate the overall detection performance. AP is defined as follows:


(19) $$\begin{array}{l} AP=\underset{n}{\sum }\left(r_{n+1}-r_{n}\right)p_{interp}\left(r_{n+1}\right) \end{array}$$

(20) $$\begin{array}{l} p_{interp}\left(r_{n+1}\right)=\underset{\tilde{r}:\tilde{r}\geq r_{n+1}}{\max}p\left(\tilde{r}\right) \end{array}$$

where $p\left(\tilde{r}\right)$ is the measured precision at recall $\tilde{r}$.

## 4. Results and discussion

### 4.1. Ablation study

**Figure 7:**
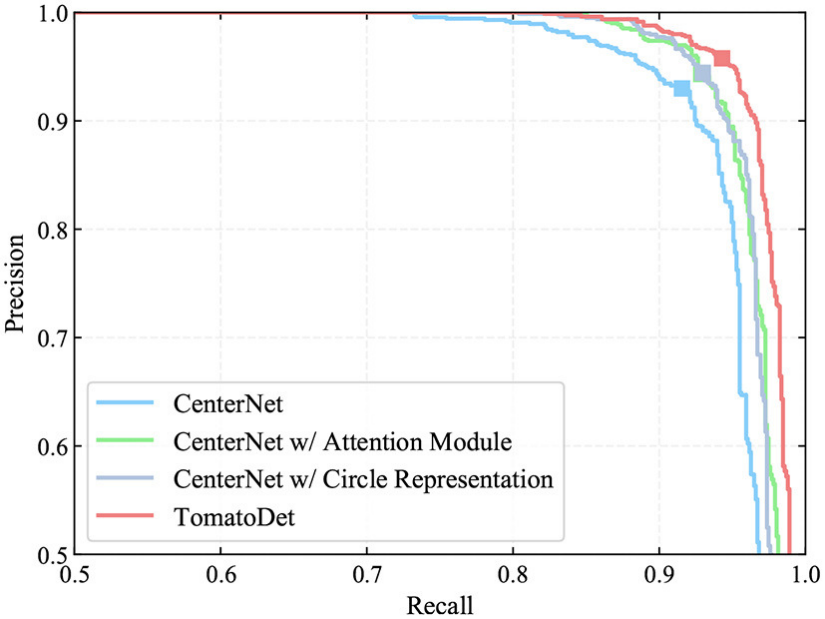
PR curves of the major components of TomatoDet for ablation study. The markers indicate the points where recall and precision are obtained when the prediction confidence threshold equals 0.6.

In this study, an attention mechanism and a circle representation are incorporated to the proposed detector. In order to evaluate the effectiveness of each component, an ablation study is performed on the tomato dataset. The results of the ablation experiments are shown in Table 2 and Figure 7.

**Table 2:** Ablation study on the major components of TomatoDet.

**Attention**	**Circle**	**Recall**	**Precision**	**F_1_**	**AP**
**module**	**representation**	**(%)**	**(%)**	**(%)**	**(%)**
		91.56	92.98	92.26	95.75
✓		92.87	94.32	93.59	97.11
	✓	92.98	94.43	93.70	96.98
✓	✓	94.30	95.77	95.03	98.16

From Table 2, we can see that the incorporation of the attention mechanism brought a significant improvement of all the indexes including the recall, precision, F_1_ score and average precision (AP). The F_1_ score and AP increases by 1.33 and 1.36%, respectively. This verifies the advantages of the proposed attentive-dla34 backbone, which optimizes the focus of the network and boosts the representation power. We also performed a contrast experiment to verify the effectiveness of the circle representation. With circle representation, the F_1_ score and AP increases by 1.44 and 1.23%, respectively, as shown in Table 2. This benefits from the intrinsic shape fitting of the new circle representation to tomatoes, which can reduce the degree of freedom of the rectangle representation and simplify the regression process from detected keypoints. We also show the precision-recall (PR) curves of different components in Figure 7. The markers indicate the points where recall and precision are obtained when the confidence threshold equals 0.6. It can be seen that the detection performance improves significantly with the incorporation of different components.

### 4.2. Comparison of different methods

To verify the performance of the proposed TomatoDet model, we designed a comparative experiment of the state-of-the-art detection algorithms, including YOLOv2 (Redmon and Farhadi, 2017), YOLOv3 (Redmon and Farhadi, 2018), YOLO-Tomato (Liu G. et al., 2020), YOLOv4 (Bochkovskiy et al., 2020), Faster R-CNN (Ren et al., 2015), CenterNet (Zhou et al., 2019), and the proposed model. Among all of these algorithms, the Faster R-CNN is a two-stage detector, and the others are one-stage detectors. Moreover, CenterNet and the proposed TomatoDet are anchor-free detectors, while the remaining are all anchor-based methods.

**Figure 8:**
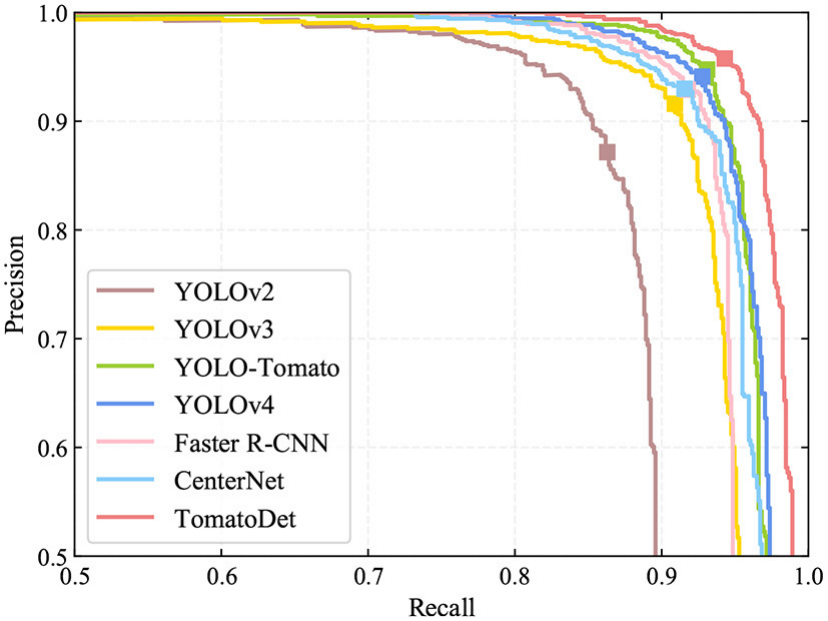
PR curves of different detection algorithms.

**Figure 9:**
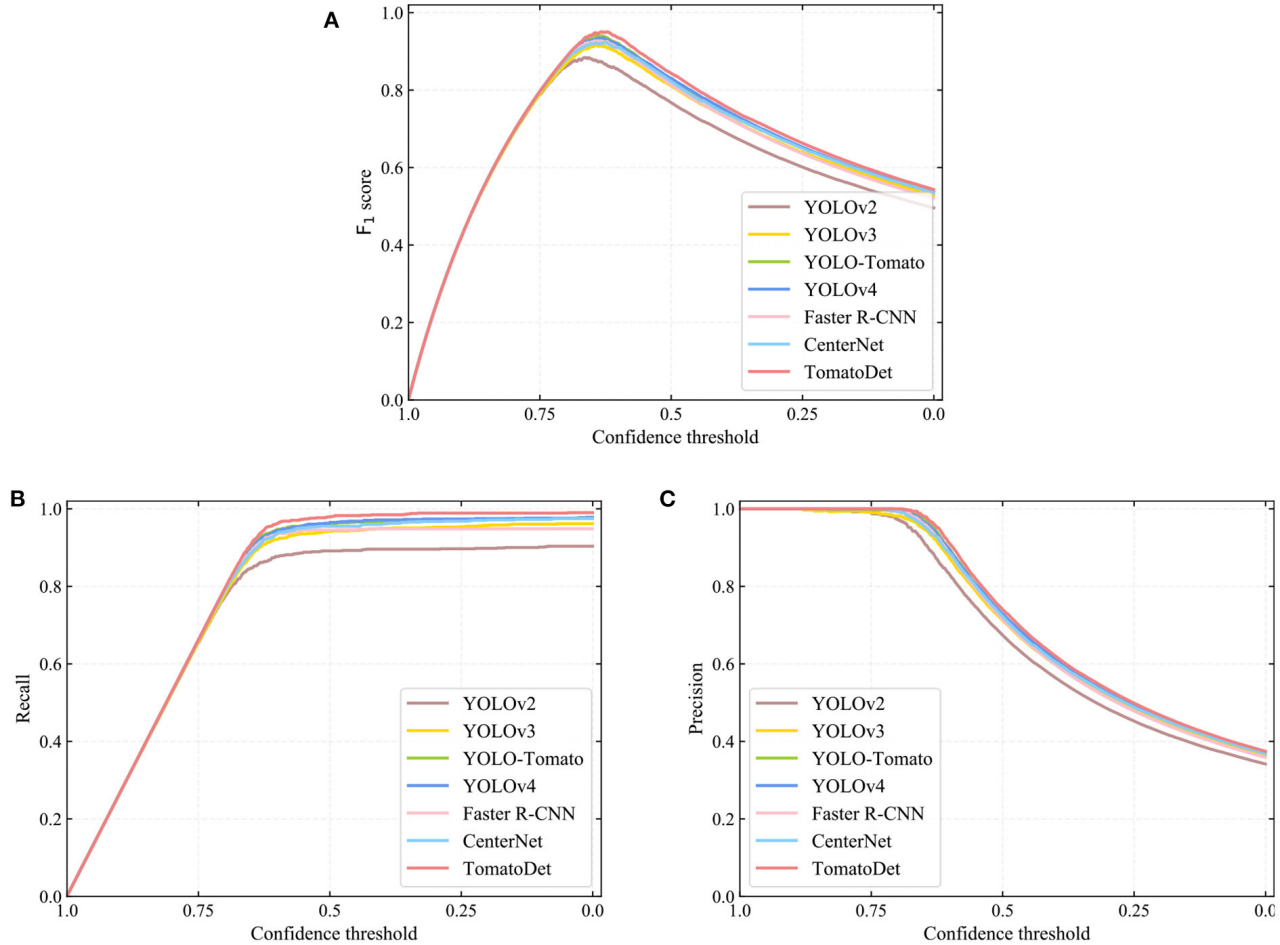
The **(A)** F_1_, **(B)** recall, and **(C)** precision curves of different detection algorithms.

The recall, precision, F_1_ score, average precision (AP), and average detection time are the evaluation indicators, as shown in Table 3. The precision-recall (PR) curves of different detection models are shown in Figure 8. In terms of detection performance, one can see that the proposed TomatoDet is superior to the other five methods. The F_1_ score of TomatoDet is 95.03%. It is 1.12% higher than that of YOLO-Tomato, which obtains the second-best performance. In terms of AP, TomatoDet performs 1.76 and 1.57% better than YOLO-Tomato and YOLOv4, respectively. Compared to CenterNet, the proposed TomatoDet is about 2.8 and 2.4% higher in terms of F_1_ score and AP, respectively. We also show the F_1_, recall and precision curves in Figure 9, separately. In accordance with the PR curves, they demonstrate the superiority of the proposed TomatoDet over other methods. This verifies the effectiveness of the proposed modifications. The introduction of CBAM guides the model to pay more attention to the ROI and thus improves the feature expression ability of the network. Besides, the adoption of bounding circles makes it easier to regress from center points to the size as the bounding circle only has one parameter, i.e., radius. Furthermore, bounding circles could match the shape of tomatoes better in nature and improve the IOU. The average detection time of the proposed model reaches 0.036 s per image. It is about 0.2 s less than Faster R-CNN and almost the same as the YOLOv2 model. The experimental results show that the proposed TomatoDet could detect tomatoes in complex environments in real-time with strong robustness.

**Table 3:** Tomato detection results of different algorithms.

**Methods**	**Recall**	**Precision**	**F_1_**	**AP**	**(ms)**
	**(%)**	**(%)**	**(%)**	**(%)**	**(ms)**
YOLOv2	86.18	87.24	86.71	88.46	30
YOLOv3	90.89	91.60	91.24	94.06	45
YOLO-Tomato	93.09	94.75	93.91	96.40	54
YOLOv4	92.76	94.11	93.43	96.59	25
Faster R-CNN	91.78	92.89	92.33	94.37	231
CenterNet	91.56	92.98	92.26	95.75	32
TomatoDet	94.30	95.77	95.03	98.16	35

### 4.3. Qualitative analysis

**Figure 10:**
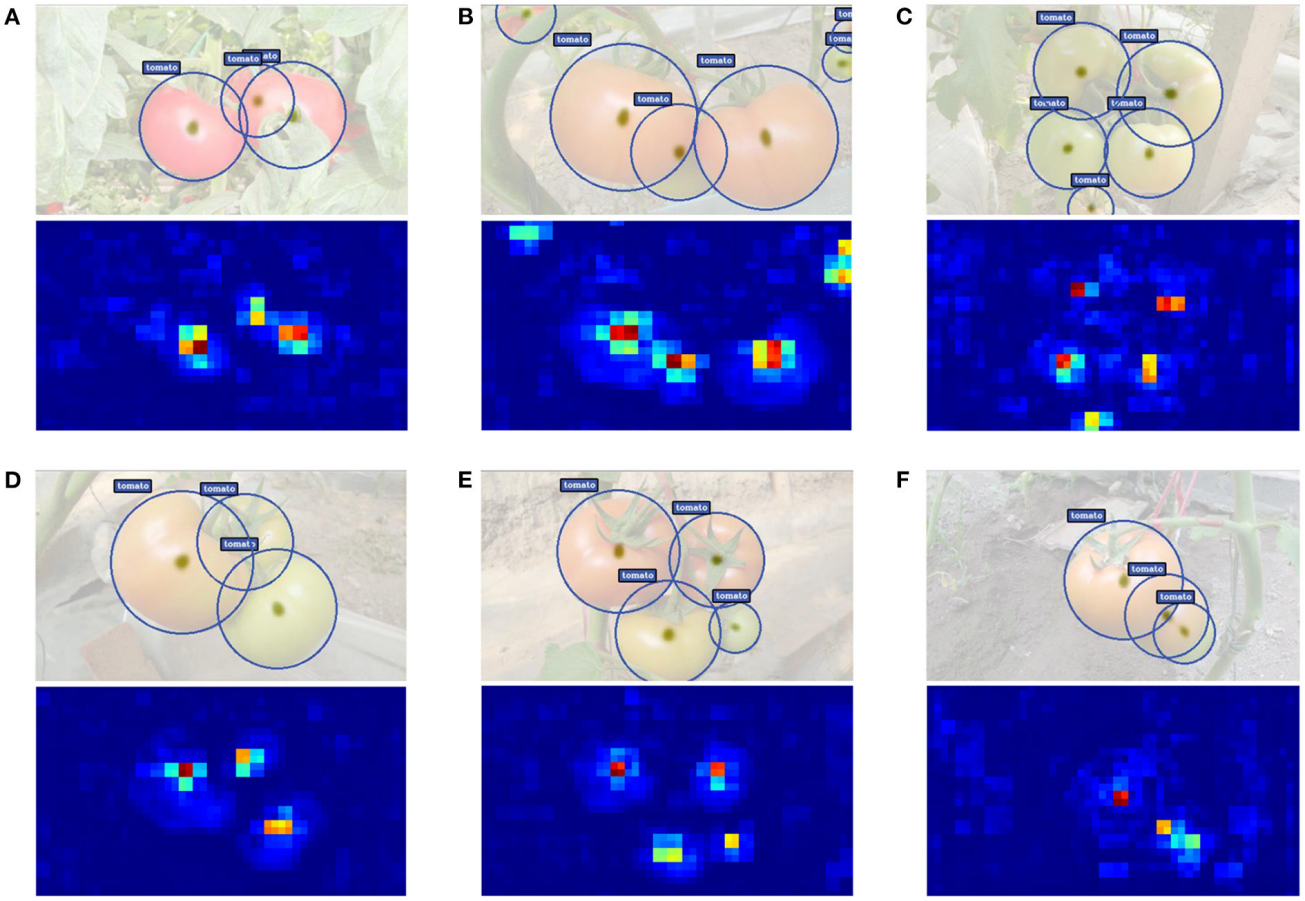
**(A–F)** Some examples of detection results along with the output heatmap.

To better understand the prediction ability of our proposed TomatoDet, the output feature is visualized. Figure 10 shows some examples of detection results along with the output heatmap. From the second row of the subfigures, one can see that through the proposed attentive-DLA34 backbone, the heatmap almost only fires at the area of tomatoes, including small and severe occluded ones. This benefits from the combination of CBAM and DLA34, which emphasizes the meaningful features throughout the network and thus boosts the representation power. Further, the keypoints for tomatoes are extracted from the peaks of the heatmap and are then regressed to the radius of the proposed bounding circle, which reduces the degree of freedom of fitting compared to the traditional bounding boxes, as is shown in the first row of the subfigures.

### 4.4. Performance of the proposed model under different lighting conditions

In the natural environment, tomatoes may be exposed to different lighting conditions due to uneven illuminations. The performance of the proposed TomatoDet under different lighting conditions is evaluated in this study. Among all the tomatoes in the test set, 425 tomatoes are in shading conditions, while 487 tomatoes are in sunlight conditions. The correct identification rate (or recall), false identification rate and missing rate are used as evaluation indicators.

**Figure 11:**
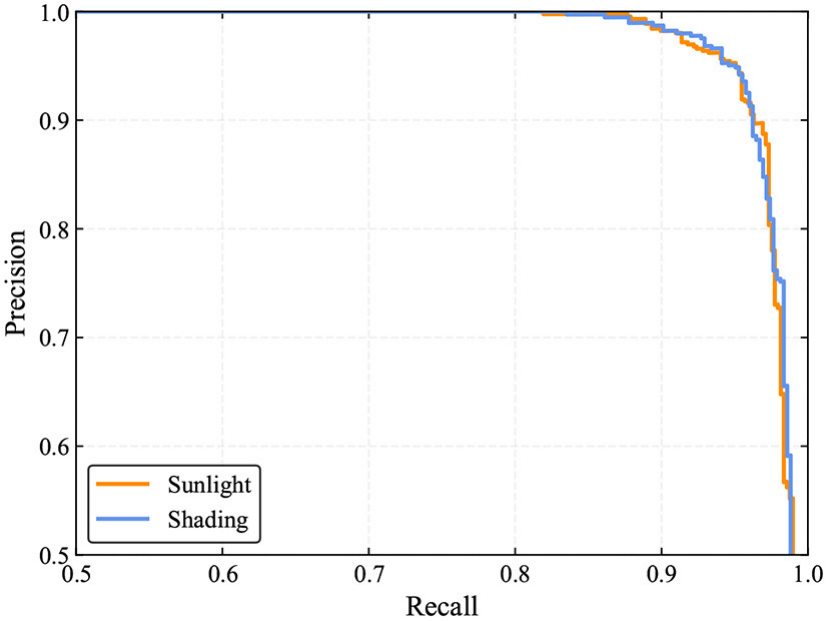
PR curves of the proposed method under different lighting conditions.

**Figure 12:**
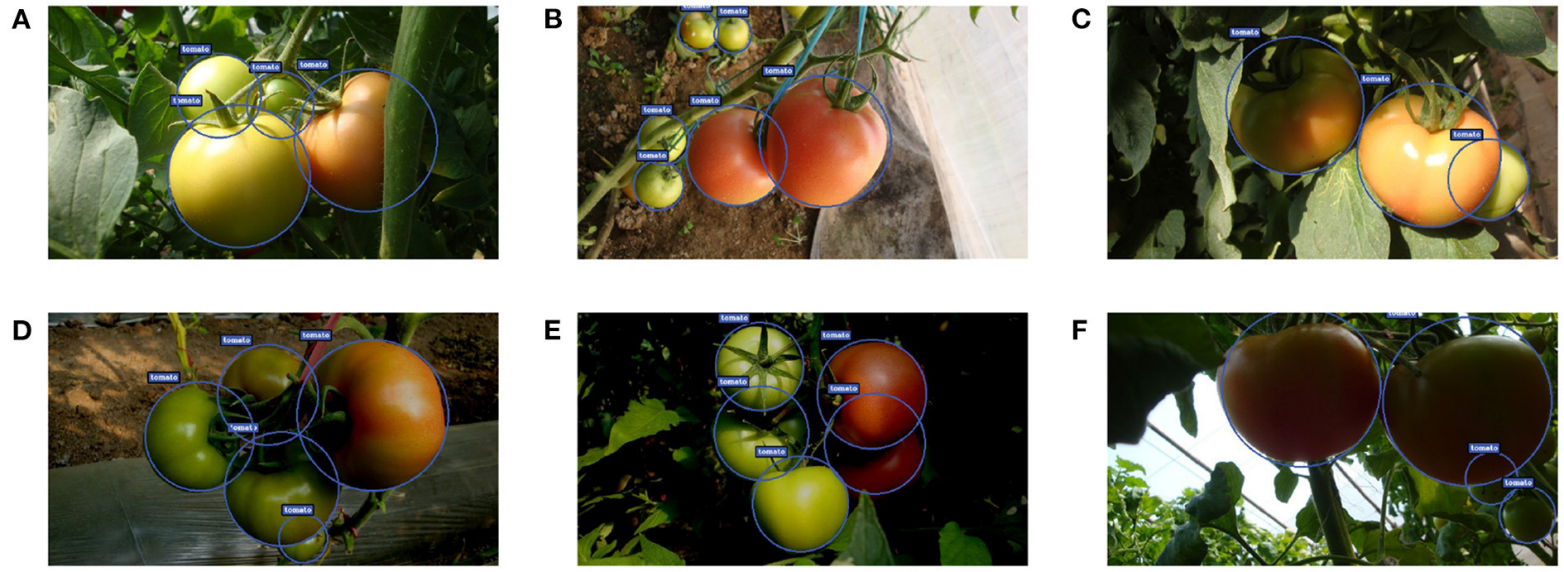
Some examples of the detection results under different lighting conditions: **(A–C)** sunlight conditions, and **(D–F)** shading conditions.

As shown in Table 4, 460 out of 487 tomatoes are correctly identified by the TomatoDet under sunlight conditions. The counterpart is 400 out of 425 for the shading conditions. The correct identification rates are comparable. The false identification rates are 4.56 and 3.85% for sunlight and shading conditions, respectively. This means that some of the detections are falsely recognized as tomatoes, which in fact are leaves, branches, or other backgrounds. This occurs when the background presents similar color and shape to tomatoes. The above results show that the proposed method is robust under different lighting conditions in real scenes. From Figure 11, one can see that the PR curves under sunlight and shading conditions are comparable, showing the robustness of the proposed method to different lighting conditions. Some examples are shown in Figure 12.

**Table 4:** Performance of the proposed TomatoDet under different lighting conditions.

**Illumination**	**Tomato count**	**Correctly identified**		**Falsely identified**		**Missed**	
		**Amount**	**Rate (%)**	**Amount**	**Rate (%)**	**Amount**	**Rate (%)**
Sunlight	487	460	94.46	22	4.56	27	5.54
Shading	425	400	94.12	16	3.85	25	5.88

### 4.5. Performance of the proposed model under different occlusion conditions

**Figure 13:**
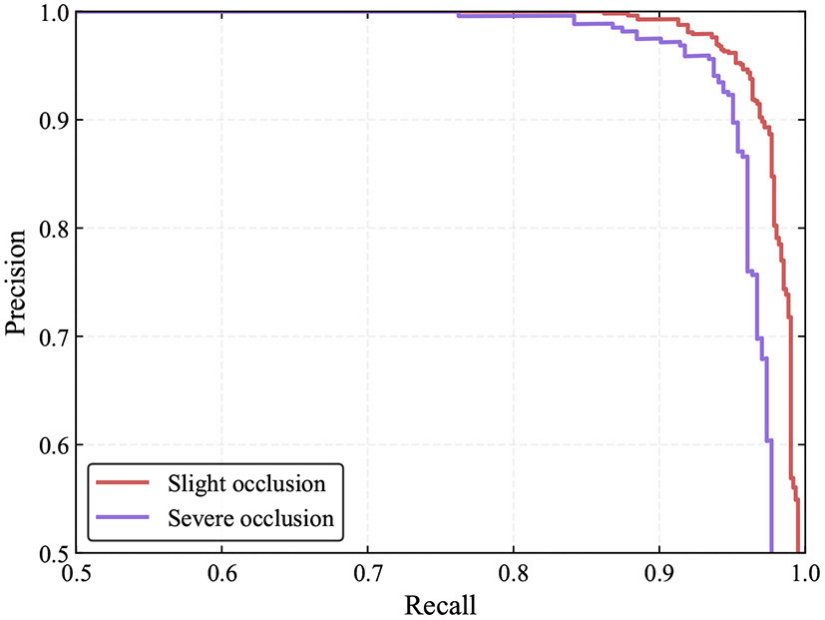
PR curves of the proposed method under different occlusion conditions.

In the greenhouse, tomatoes are inevitably obscured by leaves or branches and overlap with each other. This will have a certain impact on tomato detection. In this study, we also evaluate the performance of the proposed method under different occlusion conditions. As in YOLO-Tomato (Liu G. et al., 2020), depending on the degree of occlusion or overlap, we classify tomatoes as slight and severe occlusion cases. Severe cases refer to tomatoes being blocked by leaves, branches, or other tomatoes by more than 50% degrees. Conversely, tomatoes are regarded as slight cases. The detection results are shown in Table 5 and Figure 13.

**Table 5:** Performance of the proposed TomatoDet under different occlusion conditions.

**Occlusion** **condition**	**Tomato count**	**Correctly identified**		**Falsely identified**		**Missed**	
		**Amount**	**Rate (%)**	**Amount**	**Rate (%)**	**Amount**	**Rate (%)**
Slight case	609	576	94.58	22	3.68	33	5.42
Severe case	303	284	93.73	16	5.33	19	6.27

**Figure 14:**
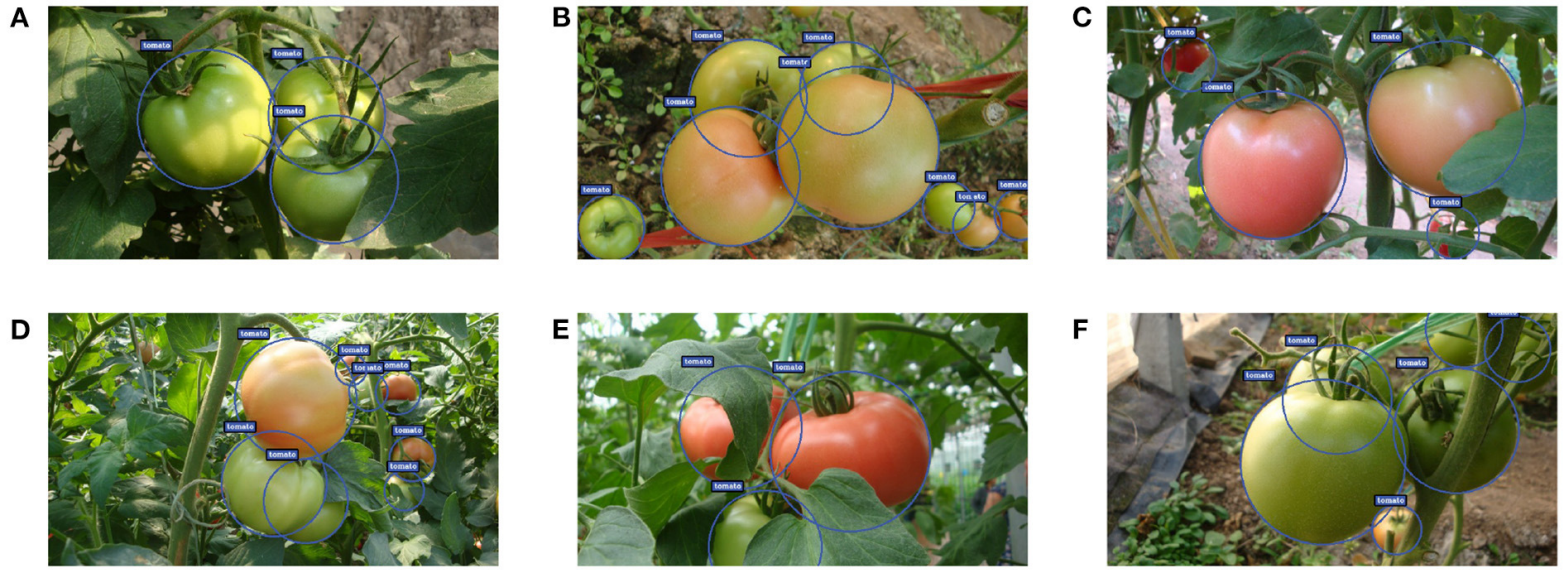
Some examples of detection results under different occlusion conditions: **(A–C)** slight cases and **(D–F)** severe cases.

Based on the above experiments, one can see that the detection performance for tomatoes under slight occlusion cases is marginally better than that of tomatoes under severe cases. This shows that occluded and overlapped tomatoes cause inaccurate detections. Nevertheless, most of the occluded and overlapped tomatoes can be detected by our model correctly. This is achieved by the accurate keypoints estimation resulting from the implicit contextual information utilization of the convolutional neural networks since the networks learn hierarchical features through multiple levels of abstraction. However, it is believed that the detection performance of occluded tomatoes can be further improved by exploiting contextual information explicitly (Liu L. et al., 2020). Figure 14 shows some examples of detection results for both cases.

## 5. Conclusions and future work

In this study, we propose TomatoDet, an improved anchor-free detector for tomato detection based on CenterNet. The proposed detector incorporates an attention mechanism to optimize the focus of the network and thus boost the representation power. In addition, a circle representation is introduced to adapt the detector to our specific detection task. With circle representation, the degree of freedom for tomato fitting is reduced and the regression process from keypoints to the size is simplified.

The experimental results show that the proposed TomatoDet is superior to other state-of-the-art detectors for tomato detection in the greenhouse. It can also detect tomatoes under different lighting and occlusion conditions with strong robustness.

Although the proposed model has achieved a good performance on the tomato datasets, there is still much space for further development. They can be summarized as follows:

When the overlap or occlusion area is high, the detection rate will drop. One possible solution is to incorporate contextual information such as branches or leaves to improve the detection accuracy.

The experimental dataset is relatively small and more data are needed for training and verification in the future study.

Moreover, the characteristics of tomatoes in different growing stages will be analyzed to realize multi-stage tomato detection.

## Data availability statement

The raw data supporting the conclusions of this article will be made available by the authors, without undue reservation.

## Author contributions

GL conceived the research idea. GL and ZH designed the methodology. JL and HL performed the experiments and analysis. GL and KL wrote the original draft. WZ and KL revised the manuscript. KL supervised the experiments. All authors contributed to the article and approved the submitted version.

## Funding

This study was supported by the Weifang Science and Technology Development Plan (2021GX054), Doctoral Research Foundation of Weifang University (2022BS70), and Natural Science Foundation of Shandong Province (ZR2021QC173).

## Conflict of interest

The authors declare that the research was conducted in the absence of any commercial or financial relationships that could be construed as a potential conflict of interest.

## Publisher's note

All claims expressed in this article are solely those of the authors and do not necessarily represent those of their affiliated organizations, or those of the publisher, the editors and the reviewers. Any product that may be evaluated in this article, or claim that may be made by its manufacturer, is not guaranteed or endorsed by the publisher.
